# Impact of the COVID-19 pandemic on lymphoma incidence and short-term survival – a Swedish Lymphoma Register Study

**DOI:** 10.2340/1651-226X.2024.35238

**Published:** 2024-04-09

**Authors:** Sara Ekberg, Daniel Molin, Simon Pahnke, Fanny Bergström, Elsa Brånvall, Karin E. Smedby, Tove Wästerlid

**Affiliations:** a Clinical Epidemiology Division, Department of Medicine Solna, Karolinska Institutet, Stockholm, Sweden; b Department of Immunology, Genetics and Pathology, Uppsala University, Uppsala, Sweden; c Department of Cancer Immunotherapy, Uppsala University, Uppsala, Sweden; d Department of Medicine, Capio St Göran Hospital, Stockholm, Sweden; e Department of Hematology, Karolinska University Hospital, Stockholm, Sweden

**Keywords:** COVID-19, pandemic, lymphoma, incidence, mortality

## Abstract

**Background & purpose:**

The COVID-19 pandemic posed a large challenge for healthcare systems across the world. Comprehensive data on the impact of the COVID-19 pandemic on incidence and mortality in lymphoma are lacking.

**Patients/methods:**

Using data from the Swedish lymphoma register, we compare incidence and 1-year survival of lymphoma patients in Sweden before (2017–2019) and during the pandemic (2020 and 2021).

**Results:**

Fewer patients were diagnosed with lymphomas during March–June 2020, but the annual incidence rates for 2020 and 2021 were similar to those of 2017–2019. A larger proportion of patients presented with stage IV disease during 2021. There were no differences in other base-line characteristics nor application of active treatment in pre-pandemic and pandemic years. One-year overall survival was not inferior among lymphoma patients during the pandemic years compared to pre-pandemic years i.e., 2017–2019.

**Interpretation:**

The COVID-19 pandemic had limited impact on the incidence and mortality of lymphoma in Sweden.

## Introduction

The emergence of the coronavirus disease 2019 (COVID-19) pandemic caused by severe acute respiratory syndrome coronavirus 2 (SARS-CoV-2), posed a large challenge for the healthcare systems across the world [1, 2]. It is known that the sudden onset of the pandemic and its demand for healthcare resources impacted diagnosis and care of many types of diseases. For example, the number of patients hospitalised for myocardial infarction decreased during initial lockdowns [3]. Further, an overall decline of all cancer types including screening sensitive cancers have been described during the initial months of the pandemic [4–7]. In a nation-wide Swedish study of all patients diagnosed with cancer, 1-year mortality increased during the pandemic, indicating that patients with cancer may have had delayed access to care [8]. Moreover, a larger proportion of patients with cancer were diagnosed at a more advanced stage [5]. In addition, active therapy for cancer has been shown to be a risk factor for COVID-19 death and need for intensive care [9].

For some lymphoma subtypes, where onset and progression of disease can occur rapidly and require prompt treatment, a delay in diagnosis may lead to a more serious disease course and increased mortality [4]. Also, patients with lymphoma have compromised immune systems both due to the lymphoma itself and the immunosuppressive treatment. Several studies have reported a more severe COVID-19 trajectory among patients with lymphoma compared to the general population and also compared to patients with solid organ cancer [10, 11].

Less data have been reported regarding the impact of the COVID-19 pandemic on lymphoma incidence. In one study no decrease in incidence was seen [4], whereas in two other studies an initial decline during the first months of the pandemic was observed [6, 7].

In this report, we aim to assess the impact of the COVID-19 pandemic on the incidence, patient characteristics, and survival of patients diagnosed with lymphoma in Sweden.

## Materials and methods

Incident lymphoma cases between 2017 and 2021 were identified using the Swedish Lymphoma register (SLR) (*n* = 10,691). The SLR records all cases of adult lymphoma in Sweden, and contains detailed clinical data on disease characteristics, prognostic indices, treatment, response, and outcomes. The coverage is 95% compared to the National Cancer Register [12], to which reporting is mandatory by law. However, there is a known lag in the registration. In 2022, for the purposes of this study, we reviewed coverage of the SLR compared with the Swedish National Cancer Register, by hospital [13]. Seven hospitals (of 52) with less than 70% of the lymphoma cases reported to the SLR for 2021, were therefore excluded from the study (*n* = 1,457 cases 2017–2021, 13%). Consequently, this study encompassed all hospitals with high coverage for 2021, covering >85% of all lymphoma cases in Sweden 2017–2021 (*n* = 9,237). Data from SLR are regularly linked to the National Cause of Death register to capture vital status and death dates.

Aggressive lymphoma subtypes were defined as diffuse large B-cell lymphoma, T-cell lymphoma, classical Hodgkin lymphoma, mantle cell lymphoma, and aggressive lymphoma not otherwise specified (NOS). Hodgkin lymphoma was classified as an aggressive lymphoma in this study due to the need for immediate curative treatment in most cases. Indolent lymphoma subtypes were defined as follicular lymphoma, lymphoplasmacytic lymphoma, marginal zone lymphoma, small lymphocytic lymphoma, nodular lymphocyte predominant Hodgkin lymphoma, and indolent lymphoma NOS. The pandemic era was defined as 2020 and 2021, and the pre-pandemic era as 2017–2019. Clinical characteristics were summarised as frequencies and compared (2020 vs. 2017–2019 and 2021 vs. 2017–2019) using the Chi-square test. Age at diagnosis was categorised (<70 years/70–84 years/≥ 85 years) and presented as median value and range (compared using the Wilcoxon rank test).

To assess the impact of the COVID-19 pandemic on lymphoma incidence, the number of incident lymphoma cases per month was plotted together with number of COVID-19 hospitalisations (publicly available at fhm.se) during the pandemic era and the mean number of incident lymphoma cases per calendar month during the pre-pandemic era.

Further, we calculated incidence rates per 100,000 person-years using data from mortality.org [14]. Poisson regression models were used to estimate incidence rate ratios (IRR) with 95% confidence intervals (CI) to compare the pandemic era with the pre-pandemic era for all lymphomas combined, stratified by aggressive/indolent lymphoma and age category.

For survival analysis, patients were followed from the date of diagnosis until death, with a maximum follow-up of 12 months. The Kaplan-Meier method was used to estimate survival proportions and Cox regression models to estimate hazard ratios (HR) with 95% CI adjusted for age category, sex, and Ann Arbor stage comparing patients diagnosed before and during the pandemic years. The proportional hazards assumption was formally tested using Schoenfeld residuals. All analyses were conducted using Stata17.

The study was approved by the Ethical Review Board in Stockholm, Sweden (2019-00242/2018/2631-31).

## Results

A total of 9,237 patients diagnosed with lymphoma in Sweden 2017–2021 were included in this study. Of these, 1,815 and 1,912 lymphoma cases were diagnosed during the pandemic years 2020 and 2021, respectively, and 5,510 were reported during the pre-pandemic period (average 1,837 cases/year).

Patient characteristics and active treatment stratified by year of diagnosis are presented in Table 1. Median age was higher at diagnosis both during 2020 (72 years) and 2021 (73 years), compared to 2017–2019 (71 years), *p* < 0.001. Patient characteristics and active treatment stratified by indolent and aggressive subtypes are presented in Supplementary Tables 1 and 2. Among all lymphomas, there was a larger proportion of patients who presented with stage IV disease during 2021 (46.1%) compared to 2017–2019 (41.6%) (*p* < 0.001). The same pattern was seen for both indolent and aggressive lymphoma subtypes (Supplementary Figure 1a–c). There were no significant differences in distribution of sex, performance status or lactate dehydrogenase nor active treatment (Supplementary Figure 2a–c) between pre-pandemic and pandemic years.

**Table 1:** Characteristics of lymphoma patients diagnosed before (2017–2019) and during (2020 and 2021) the COVID-19 pandemic.

Patient characteristics	Year of diagnosis					
	2017–2019		2020		2021	
Total	5,510		1,815		1,912	
Median age (IQ range)	71 (60, 78)		72 (59, 79)		73 (62, 80)	
	*n*	%	*n*	%	*n*	%
**Age, categorised (years)**						
<70	2,485	45.2	820	45.2	778	40.7
70–85	2,585	47.0	856	47.2	963	50.4
>85	430	7.8	139	7.7	171	8.9
**Sex**						
Male	3,225	58.5	1,047	57.7	1,135	59.4
Female	2,285	41.5	768	42.3	777	40.6
**Performance status**						
Asymptomatic	3,018	54.8	965	53.2	1,010	52.8
Ambulatory	1,601	29.1	528	29.1	573	30.0
Bedbound <50%	409	7.4	137	7.5	143	7.5
Bedbound >50%	235	4.3	97	5.3	86	4.5
Bedbound	127	2.3	39	2.1	51	2.7
Missing	120	2.2	49	2.7	49	2.6
**Stage**						
Ann Arbor I	1,075	19.5	322	17.7	327	17.1
Ann Arbor II	822	14.9	271	14.9	249	13.0
Ann Arbor III	843	15.3	261	14.4	265	13.9
Ann Arbor IV	2,301	41.8	769	42.4	882	46.1
Missing	469	8.5	192	10.6	189	9.9
**Elevated LDH**						
No	2,883	52.3	938	51.7	1,021	53.4
Yes	2,312	42.0	758	41.8	787	41.2
Missing	315	5.7	119	6.6	104	5.4
**Subtype, grouped**						
Aggressive subtypes	3,055	58.8	1,019	60.3	982	55.9
Indolent subtypes	2,138	41.2	672	39.7	775	44.1
**Active Treatment**						
No	1,546	28.1	519	28.6	590	30.9
Yes	3,813	69.2	1,285	70.8	1,312	68.6
Missing	151	2.7	11	0.6	10	0.5
**Subtype**						
DLBCL	1,753	31.8	584	32.2	566	29.6
TCL	375	6.8	126	6.9	109	5.7
HL	457	8.3	152	8.4	157	8.2
MCL	342	6.2	108	6.0	121	6.3
Aggressive NOS	128	2.3	49	2.7	29	1.5
FL	772	14.0	245	13.5	282	14.8
LPL	360	6.5	125	6.9	142	7.4
MZL	430	7.8	148	8.2	165	8.6
SLL	157	2.8	30	1.7	50	2.6
NLPHL	38	0.7	16	0.9	7	0.4
Indolent NOS	381	6.9	108	6.0	129	6.8
Other	316	5.7	123	6.8	153	8.0

LDH: Lactate dehydrogenase; DLBCL: Diffuse Large B-cell Lymphoma; TCL: T-cell Lymphoma; HL: Hodgkin Lymphoma; MCL: Mantle cell lymphoma; FL: Follicular lymphoma; LPL: lymphoplasmacytic lymphoma; MZL: Marginal Zone Lymphoma; SLL: Small Lymphocytic Lymphoma; NLPHL: Nodular Lymphocyte Predominant Hodgkin Lymphoma; NOS: Not otherwise specified; IQ: Interquartile.

**Figure 1:**
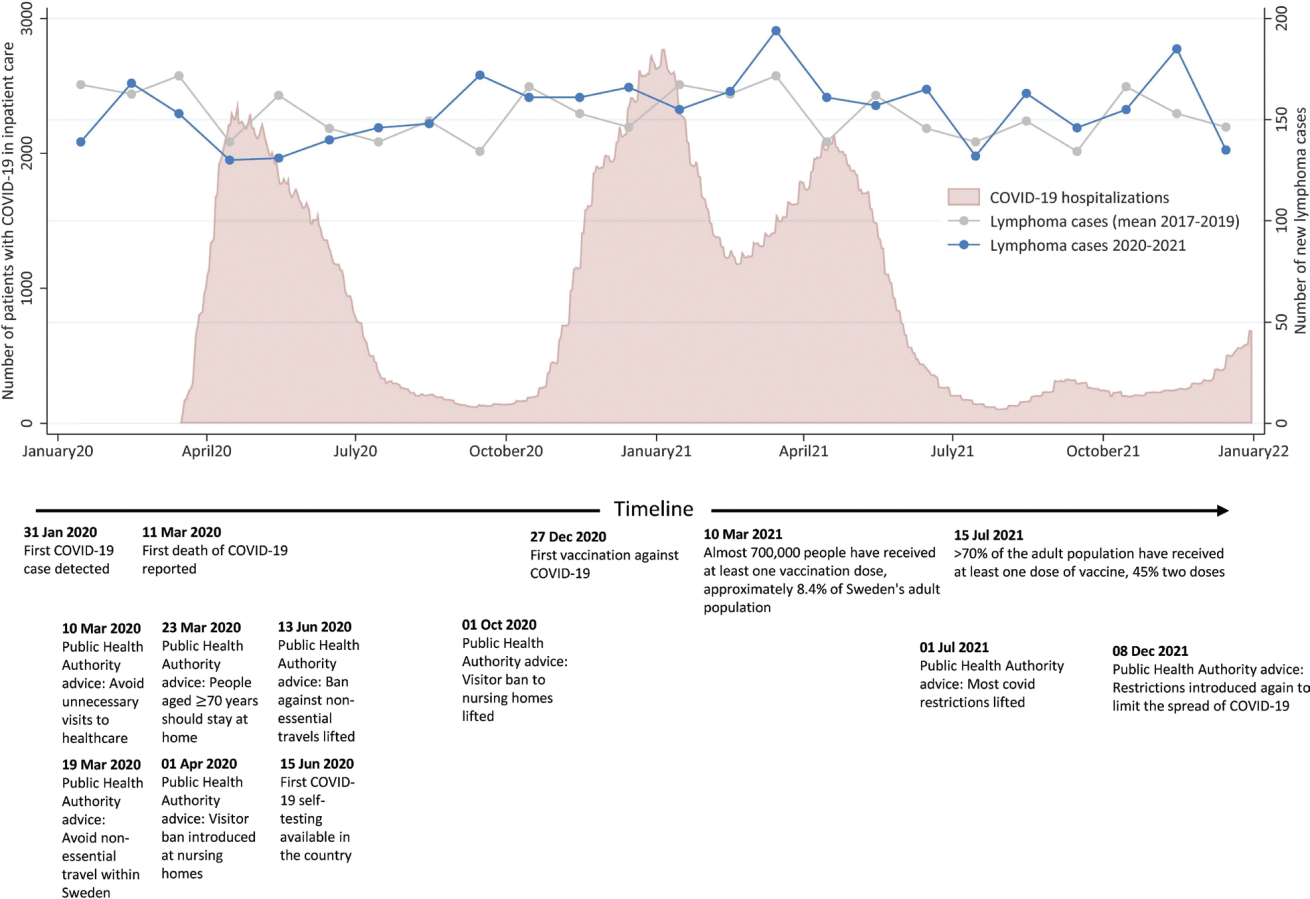
The number of new lymphoma cases reported to the Swedish Lymphoma Register per calendar month during 2020 and 2021 compared with the mean number of lymphoma cases reported per calendar month in 2017–2019. The number of patients with COVID-19 in inpatient care (including intensive care units), together with selected events, advice, and regulations connected to the COVID-19 pandemic handling in Sweden, are also presented.

The number of new lymphoma cases per calendar month during 2020 and 2021 contrasted to the mean number of lymphomas reported per calendar month in 2017–2019 is presented in Figure 1. Here, we see a consistent numerical drop in the number of reported cases of lymphoma compared to 2017–2019 from March to June 2020. For the rest of the time period, the number of newly diagnosed cases fluctuated month by month, similar to 2017–2019. On the annual level, the overall incidence rates in 2020 and 2021 are comparable to those in 2017–2019 (IRR 2020 vs. 2017–2019: 0.97 (95%CI: 0.92, 1.02) and IRR 2021 vs. 2017–2019: 1.02 (95%CI: 0.97, 1.07)).

Stratified by indolent and aggressive subtypes, we see that the lower number of lymphoma diagnoses during the start of the pandemic may have been driven by a lower number of indolent cases whereas the reported number of aggressive subtypes lie close to the mean value. The overall IRR for indolent lymphomas in 2020 compared to 2017–2019 was 0.93 (95%CI: 0.85, 1.01), which increased to 1.06 (95%CI: 0.98, 1.16) in 2021. Corresponding IRRs for aggressive subtypes were 0.98 (95%CI: 0.92, 1.06) for 2020 and 0.94 (95%CI: 0.88, 1.01) in 2021 (Supplementary Table 3). Accordingly, indolent subtypes accounted for a lower proportion of diagnosed lymphomas in 2020 (39.7%) vs. 44.1% in 2021, *p* = 0.009) (Table 1).

**Figure 2:**
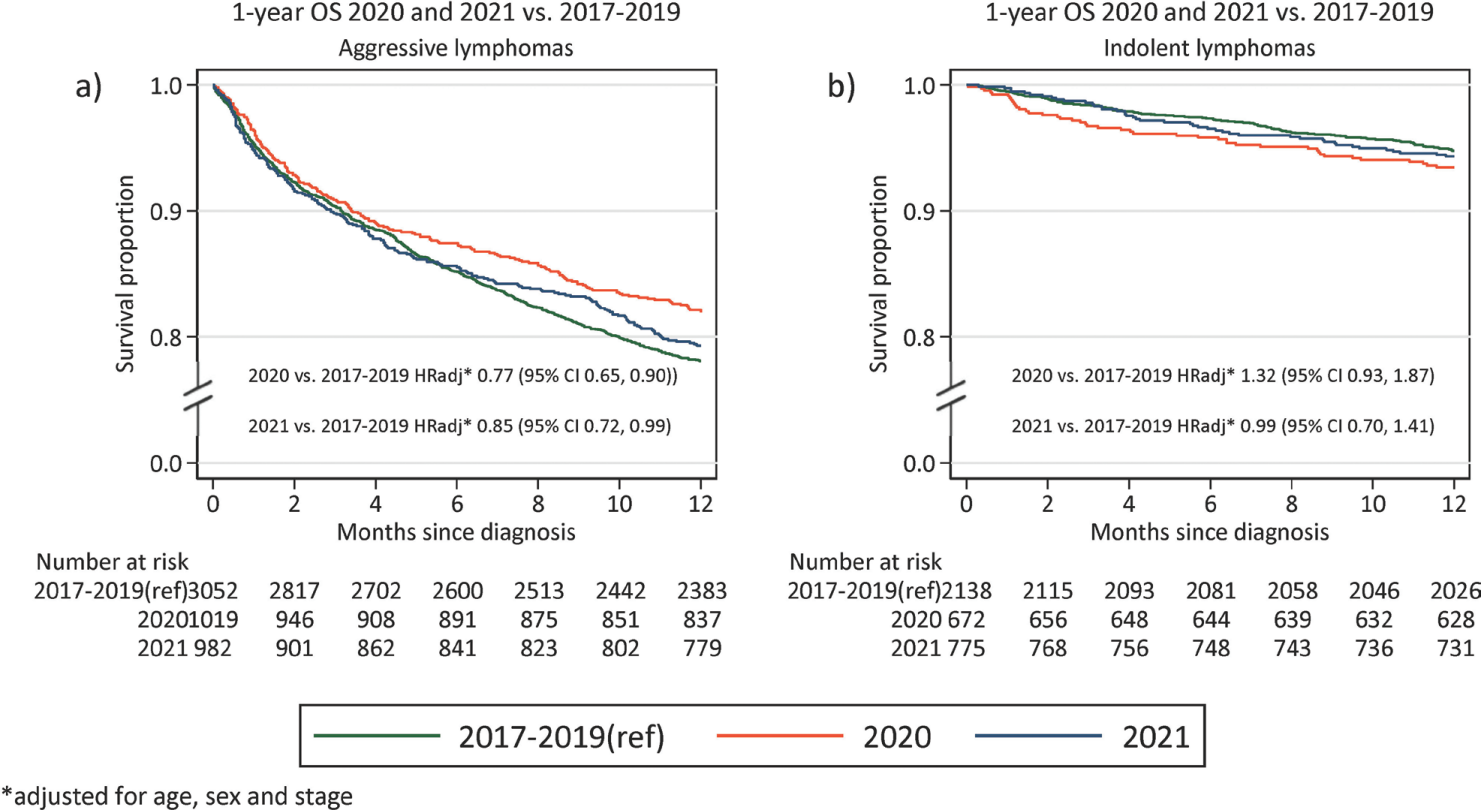
One-year overall survival (OS) for patients diagnosed in 2020 and 2021, compared to 2017–2019 (a) Aggressive lymphomas (b) Indolent lymphomas. Adjusted HR rates for mortality presented in the bottom of the graph.

Finally, we compared 1-year overall survival (OS) of patients with lymphoma diagnosed 2020–2021 to lymphoma patients diagnosed 2017–2019. One-year OS for patients with aggressive lymphoma diagnosed 2017–2019 was 78% (95% CI: 77–80%) compared to 82% (95% CI: 79–84%) and 79% (95% CI: 77–82%) for patients diagnosed 2020 and 2021, respectively (Figure 2). Adjusting for age, sex, and stage, a slightly superior OS was observed for patients diagnosed during 2020 and 2021 compared to 2017–2019 (HR 2020: 0.77 [95%CI: 0.65–0.90] and HR 2021: 0.85 [95%CI: 0.72–0.99]). There was no difference in 1-year OS for patients diagnosed with indolent lymphoma diagnosed 2020 (OS:93%, 95% CI: 91–95%) and 2021 (OS:94% (95% CI: 92–96%), compared to patients diagnosed 2017–2019 (OS 95% [95% CI: 94–96%], adjusted HR 2020: 0.93 [95% CI: 0.93–1.87] and HR 2021: 0.99 [95% CI: 0.70–1.41]) (Figure 2).

## Discussion

We describe the impact of the COVID-19 pandemic on incidence, mortality and patient characteristics of patients with lymphoma in Sweden. Overall, the impact of the COVID-19 pandemic on incidence and mortality in lymphoma in Sweden appeared to be small. However, we demonstrate a consistently lower number of patients diagnosed with lymphoma from the onset of the pandemic in March–June 2020. The drop in reported cases of lymphoma during this period seems to be primarily driven by a lower number of diagnosed indolent lymphomas whereas the number of patients diagnosed with aggressive lymphomas remained similar. After this initial dip, we see consistent incidence rates but slightly altered patient characteristics with a higher median age at diagnosis and larger proportion of patients with stage IV disease during the pandemic years. Despite this, we did not observe increased lymphoma mortality during the pandemic years.

The consistently lower number of registered lymphoma patients March–June 2020 likely represent an effect of the onset of the pandemic. Although no previous study has focussed solely on lymphoma, similar declines in diagnostic rates of lymphoma during the initial months of the pandemic have been reported [6, 7]. Although Sweden’s COVID strategy differed from other countries and there were no strict lock-downs during the beginning of the pandemic [1, 2], there was a general knowledge that the situation in the hospitals was strained. Therefore, it is likely that patients who did not have an imminent need to seek healthcare may have delayed doing so. It is plausible that this should predominantly affect indolent lymphomas as patients with aggressive lymphomas are more often immediately symptomatic. Regardless, there was a higher proportion of patients with both aggressive and indolent lymphoma who presented with stage IV disease diagnosed in 2021, potentially indicating delayed contact with health care.

We do not demonstrate increased mortality during 2020–2021 compared to 2017–2019. This is reassuring although a limitation of the present study is the short follow-up time. Importantly, with only 1-year OS reported we are likely unable to measure the potential negative impact of a higher proportion of stage IV disease at diagnosis, which may appear with increased follow-up time. Our results are in contrast with those from a regional study in Canada that showed decreased survival of lymphoma patients during 2020 [4]. Rather, we observe a slightly superior 1-year OS for patients diagnosed with aggressive lymphoma in 2020 and 2021 compared to 2017–2019. This finding should be interpreted with caution as the difference is small and could be due to other unmeasured differences by calendar year or improved survival over time, although first line treatment guidelines did not change in Sweden between 2017 and 2021, and we do not expect to capture improvement in survival due to novel agents in later lines through 1-year OS. However, if true, it could potentially indicate a higher caution among treated patients, which may have lowered the risk of treatment related deaths. Also, although most relapses for aggressive lymphoma occur within 2 years, the follow-up time of 1 year precludes us from capturing some relapses of aggressive lymphoma. For patients with indolent lymphoma, survival was similar in pre-pandemic and pandemic years. As COVID-19 related mortality has been reported to be higher among patients with lymphoma [10, 15], the stable survival rates observed in the present study during the pandemic years are reassuring. Unfortunately, detailed treatment data was not available in the present study but administration of active treatment did not significantly differ between pandemic and pre-pandemic years and the stable survival rates indicate that potentially altered or delayed treatment has not impacted short-term survival. This is important information both when planning for future potential pandemics and for patients with lymphoma.

To conclude, we observe a lower number of newly diagnosed patients with lymphoma in the first months following the onset of the pandemic but annual incidence rates remained similar. A slightly larger proportion of patients presented with stage IV disease during 2020 and 2021 but with 1-year follow-up we did not observe increased mortality among patients with lymphoma during the pandemic years. The impact of the COVID-19 pandemic on the incidence and mortality of lymphoma in Sweden thus appears to have been limited.

## Supplementary Material

Impact of the COVID-19 pandemic on lymphoma incidence and short-term survival – a Swedish Lymphoma Register Study

## Data Availability

Study data are available upon request if in line with ethical and legal permissions.
