# Supplementary material for: Impact of the COVID-19 pandemic on lymphoma incidence and short-term survival – a Swedish Lymphoma Register Study
Source: Acta Oncol. 2024 Apr 9;63:35238. doi: 10.2340/1651-226X.2024.35238 (PMC11332491; doi:10.2340/1651-226X.2024.35238)
Supplement: Impact of the COVID-19 pandemic on lymphoma incidence and short-term survival – a Swedish Lymphoma Register Study [file AO-63-35238-s1.pdf]

Supplementary material has been published as submitted. It has not been copyedited or typeset by Acta Oncologica.

Supplementary Material

Supplementary Table 1. Characteristics of lymphoma patients diagnosed with aggressive lymphoma subtypes before (2017-2019) and during (2020 and 2021) the COVID-19 pandemic.

	**Year of diagnosis**					
	**2017-2019 N(%)**		**2020 N(%)**		**2021 N (%)**	
**Total**	3,055		1,019		982	
**Median age (IQ range)**	70	(58,78)	71	(56,79)	72	(59,79)
**Age, categorized (years)**						
<70	1,441	(47.2)	478	(46.9)	417	(42.5)
70-85	1,371	(45.0)	466	(45.7)	479	(48.8)
>85	238	(7.8)	75	(7.4)	86	(8.8)
**Sex**						
Male	1,860	(60.9)	600	(58.9)	605	(61.6)
Female	1,195	(39.1)	419	(41.1)	377	(38.4)
**Performance status**						
Asymptomatic	1,422	(46.5)	477	(46.8)	435	(44.3)
Ambulatory	971	(31.8)	313	(30.7)	318	(32.4)
Bedbound <50%	304	(10.0)	100	(9.8)	101	(10.3)
Bedbound >50%	189	(6.2)	76	(7.5)	67	(6.8)
Bedbound	107	(3.5)	26	(2.6)	43	(4.4)
Missing	62	(2.0)	27	(2.6)	18	(1.8)
**Stage**						
Ann Arbor I	566	(18.5)	160	(15.7)	149	(15.2)
Ann Arbor II	589	(19.3)	188	(18.4)	167	(17.0)
Ann Arbor III	512	(16.8)	158	(15.5)	152	(15.5)
Ann Arbor IV	1,188	(38.9)	406	(39.8)	409	(41.6)
Missing	200	(6.5)	107	(10.5)	105	(10.7)
**Elevated LDH**						
No	1,253	(41.0)	421	(41.3)	416	(42.4)
Yes	1,636	(53.6)	537	(52.7)	526	(53.6)
Missing	166	(5.4)	61	(6.0)	40	(4.1)
**Active Treatment**						
No	362	(11.9)	109	(10.7)	124	(12.6)
Yes	2,611	(85.5)	901	(88.4)	851	(86.7)
Missing	82	(2.7)	9	(0.9)	7	(0.7)
**Subtype**						
DLBCL	1,753	(57.4)	584	(57.3)	566	(57.6)
T-cell	375	(12.3)	126	(12.4)	109	(11.1)
HL	457	(15.0)	152	(14.9)	157	(16.0)
MCL	342	(11.2)	108	(10.6)	121	(12.3)

Aggressive NOS	128	(4.2)	49	(4.8)	29	(3.0)

DLBCL=Diffuse Large B-cell Lymphoma, TCL= T-cell Lymphoma, HL = Hodgkin Lymphoma, MCL= Mantle cell lymphoma, NOS= Not otherwise specified IQ= Interquartile

Supplementary Table 2. Characteristics of lymphoma patients diagnosed with indolent lymphoma subtypes before (2017-2019) and during (2020 and 2021) the COVID-19 pandemic.

	**Year of diagnosis**					
	**2017-2019 N (%)**		**2020 N (%)**		**2021 N (%)**	
**Total**	2,138		672		775	
**Median age (IQ range)**	71	(61,78)	72	(61,78)	73	(63,80)
**Age, categorized (years)**						
<70	953	(44.7)	302	(44.9)	304	(39.2)
70-85	1,029	(48.2)	320	(47.6)	408	(52.6)
>85	151	(7.1)	50	(7.4)	63	(8.1)
**Sex**						
Male	1,184	(55.4)	377	(56.1)	437	(56.4)
Female	954	(44.6)	295	(43.9)	338	(43.6)
**Performance status**						
Asymptomatic	1,451	(67.9)	440	(65.5)	498	(64.3)
Ambulatory	527	(24.6)	177	(26.3)	212	(27.4)
Bedbound <50%	82	(3.8)	30	(4.5)	30	(3.9)
Bedbound >50%	27	(1.3)	12	(1.8)	11	(1.4)
Bedbound	11	(0.5)	3	(0.4)	2	(0.3)
Missing	40	(1.9)	10	(1.5)	22	(2.8)
**Stage**						
Ann Arbor I	458	(21.4)	146	(21.7)	146	(18.8)
Ann Arbor II	196	(9.2)	71	(10.6)	69	(8.9)
Ann Arbor III	301	(14.1)	93	(13.8)	103	(13.3)
Ann Arbor IV	983	(46.0)	292	(43.5)	390	(50.3)
Missing	200	(9.4)	70	(10.4)	67	(8.6)
**Elevated LDH**						
No	1,462	(68.4)	452	(67.3)	530	(68.4)
Yes	555	(26.0)	173	(25.7)	192	(24.8)
Missing	121	(5.7)	47	(7.0)	53	(6.8)
**Active Treatment**						
No	1,039	(48.6)	355	(52.8)	407	(52.5)
Yes	1,044	(48.8)	316	(47.0)	367	(47.4)
Missing	55	(2.6)	1	(0.2)	1	(0.1)
**Subtype**						
FL	772	(36.1)	245	(36.5)	282	(36.4)
LPL	360	(16.8)	125	(18.6)	142	(18.3)
MZL	430	(20.1)	148	(22.0)	165	(21.3)
SLL	157	(7.3)	30	(4.5)	50	(6.5)
NLPHL	38	(1.8)	16	(2.4)	7	(0.9)
Indolent, NOS	381	(17.8)	108	(16.1)	129	(16.6)

FL = Follicular lymphoma, LPL = lymphoplasmacytic lymphoma, MZL= Marginal Zone Lymphoma SLL=Small Lymphocytic Lymphoma, NLPHL=Nodular Lymphocyte Predominant Hodgkin Lymphoma, NOS= Not otherwise specified IQ= Interquartile

Supplementary Table 3. Incidence rates of lymphoma per 100 000 person-years before (2017-2019), and during (2020 and 2021) the COVID-19 pandemic, and incidence rate ratios (IRR) with 95% confidence intervals (CI), comparing the pandemic years to the pre-pandemic period (reference) by type of lymphoma and age.

	Incidence rate per 100,000 person-years			IRR (95% CI)		
	2017-2019	2020	2021	2017-2019	2020	2021
All Lymphomas	22.9	22.3	23.3	1 (ref)	0.97 (0.92, 1.02)	1.02 (0.97, 1.07)
Age<70	12.6	12.4	11.7	1 (ref)	0.98 (0.91, 1.06)	0.93 (0.86, 1.01)
Age 70-84	69.9	63.9	72.1	1 (ref)	0.91 (0.84, 0.99)	1.03 (0.96, 1.11)
Age 85+	67.9	71.8	78.1	1 (ref)	1.06 (0.90, 1.25)	1.15 (0.98, 1.35)
Aggressive	12.7	12.5	12.0	1 (ref)	0.98 (0.92, 1.06)	0.94 (0.88, 1.01)
Age<70	7.3	7.2	6.3	1 (ref)	0.99 (0.89, 1.09)	0.86 (0.77, 0.96)
Age 70-84	37.0	34.3	35.7	1 (ref)	0.92 (0.83, 1.03)	0.96 (0.87, 1.34)
Age 85+	37.4	41.1	40.0	1 (ref)	1.10 (0.88, 1.37)	1.07 (0.85, 1.33)
Indolent	8.9	8.2	9.5	1 (ref)	0.93 (0.85, 1.01)	1.06 (0.98, 1.16)
Age<70	4.8	4.6	4.6	1 (ref)	0.94 (0.83, 1.07)	0.94 (0.83, 1.07)
Age 70-84	28.0	24.2	30.7	1 (ref)	0.87 (0.76, 0.98)	1.10 (0.98, 1.23)
Age 85+	23.6	24.3	28.6	1 (ref)	1.03 (0.78, 1.37)	1.21 (0.93, 1.58)

Supplementary Figure 1a-c. The proportion of patients with stage I, II, III and IV per 6-months 2017-2021 for a) all lymphoma patients, b) aggressive lymphomas and c) Indolent lymphomas

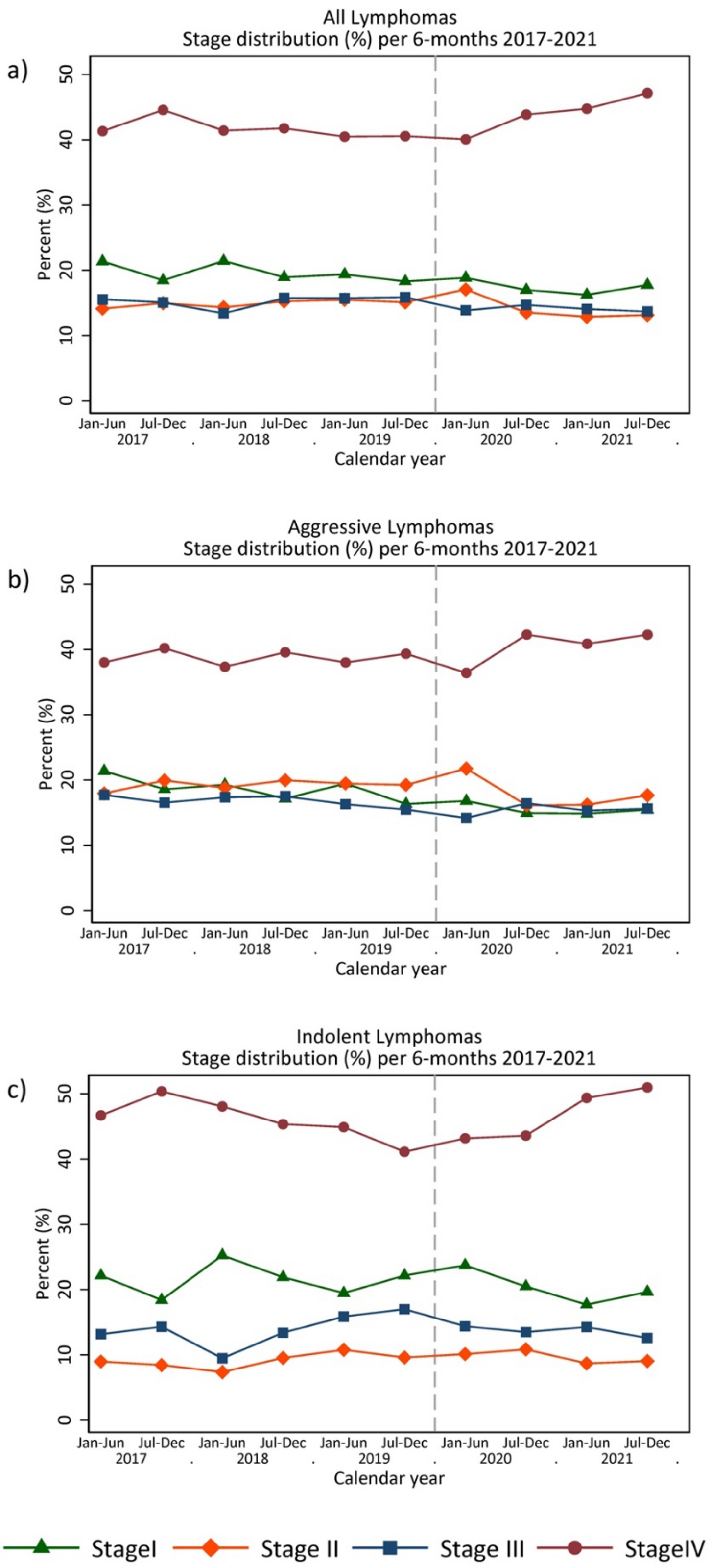

*The dashed line marks the beginning of the pandemic year 2020

Supplementary Figure 2a-c. The proportion of patients with active treatment, no active treatment and missing per 6-months 2017-2021 for a) all lymphoma patients, b) aggressive lymphomas and c) Indolent lymphomas

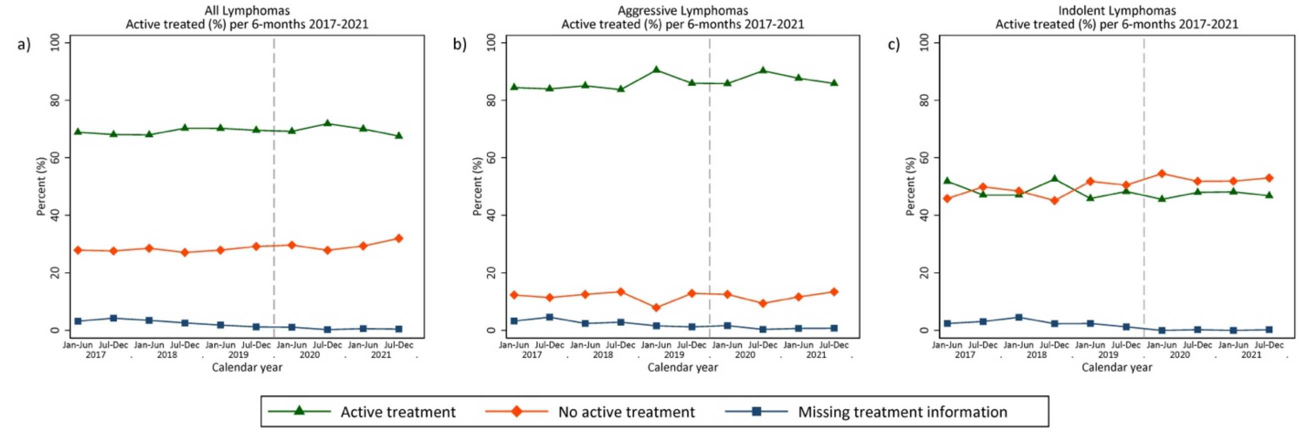

*The dashed line marks the beginning of the pandemic year 2020
